# Arthroplasty for post-traumatic carpometacarpal joint (CMC) V osteoarthritis with minced cartilage: a case report

**DOI:** 10.1515/iss-2025-0013

**Published:** 2025-07-16

**Authors:** Vincent März, Martynas Tamulevicius, Peter Maria Vogt, Frederik Schlottmann

**Affiliations:** Department of Plastic, Aesthetic, Hand and Reconstructive Surgery, Hannover Medical School, Hannover, Germany

**Keywords:** cartilage, hand, minced cartilage, osteoarthritis

## Abstract

**Objectives:**

Fractures and dislocations of the bases of the fourth and fifth metacarpal bones are rare, accounting for <1 % of all hand injuries. If left untreated or inadequately fixed, there is a risk of persistent pain, functional impairment, and post-traumatic osteoarthritis. While common surgical approaches such as arthrodesis or suspension arthroplasty have been described, data on alternative treatment options remain limited.

**Case presentation:**

This case report presents the first documented use of minced cartilage for treating post-traumatic CMC V osteoarthritis. A 17-year-old patient experienced persistent pain following fracture fixation with K-wires. After debriding arthrotomy, cartilage was harvested from the hamate bone, minced, and combined with platelet rich plasma before implantation. One year postoperatively, the patient showed improved joint space, complete pain relief, and restored hand function.

**Conclusions:**

Preliminary results suggest that minced cartilage may offer a promising alternative, but further studies are needed to confirm its long-term efficacy.

## Introduction

The carpometacarpal (CMC) joints play a crucial role in the biomechanics and overall function of the hand. Fractures and dislocations of the bases of the fourth and fifth metacarpal bones are rare injuries of the metacarpus, with an incidence of less than 1 % of all hand injuries [1]. Fractures involving the ulnar base of the fifth metacarpal may require closed fixation with K-wires or open reduction followed by immobilization [2]. When these injuries are recognized early, clinical outcomes are generally satisfactory in terms of range of motion and pain. However, if diagnosis is delayed, treatment initiated late, or reduction and/or fixation is inadequate, there is a risk of persistent pain, limited hand function, and development of post-traumatic osteoarthritis [3], 4]. Currently, only limited data are available on the treatment of post-traumatic osteoarthritis in the CMC joint area. Individual case reports describe surgical procedures such as arthrodesis of the CMC joints IV and V, suspension arthroplasty by suspending the fifth metacarpal to the shaft of the fourth metacarpal, or interposition arthroplasty using capsular tissue [5], [6], [7]. The following case report presents a novel treatment concept for CMC V joint osteoarthritis using minced cartilage.

## Case presentation

**Figure 1:**
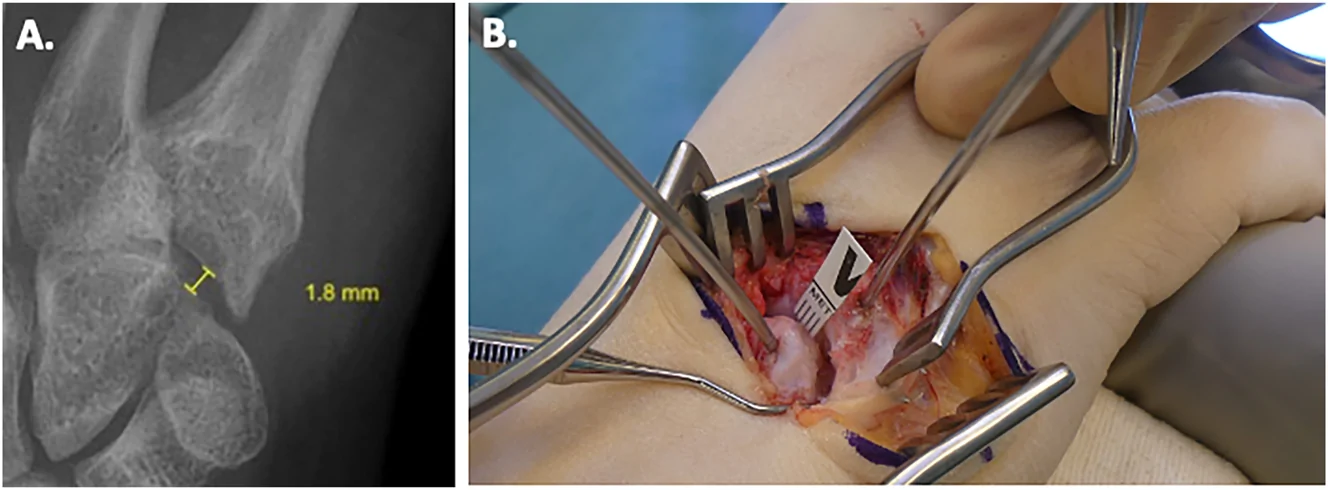
Preoperative imaging and intraoperative findings. (A) Preoperative X-ray image showing post-traumatic CMC-V arthrosis (joint space measured at 1.8 mm). (B) Intraoperative view of CMC-V osteoarthritis with central grade IV cartilage damage and osteophytic attachments.

As a result of a cycling accident, a 17-year-old left-handed male patient sustained a fracture of the right CMC V base with corresponding dislocation on the ulnar side of the base. Initial treatment involved immobilization in a plaster splint, but due to increasing dislocation, closed reduction and K-wire fixation with stabilization of the base to the hamate bone were performed. The patient presented to our clinic 1 year after the initial surgery with persistent complaints in the CMC V joint area. He reported pain during wrist hyperextension, axial compression in the CMC joint, and end-range fist closure, accompanied by a decrease in grip strength (25 kg using a hydraulic Jamar hand dynamometer, compared to 40 kg on the unaffected side). Pain intensity was rated at up to six out of 10 on the visual analog scale (VAS). He showed impairments in activities of daily living with a preoperative Disability of Arm, Shoulder and Hand (DASH) score of 25 points. With X-ray and computer tomography evidence of post-traumatic osteoarthritis of the CMC V joint (joint space measured at 1.8 mm in X-ray imaging, Figure 1A) and persistent pain under stress, surgical arthroplasty was indicated. After dorsal arthrotomy of the CMC V joint, advanced CMC V osteoarthritis with central grade IV cartilage damage and osteophytic formations was revealed (Figure 1B).

**Figure 2:**
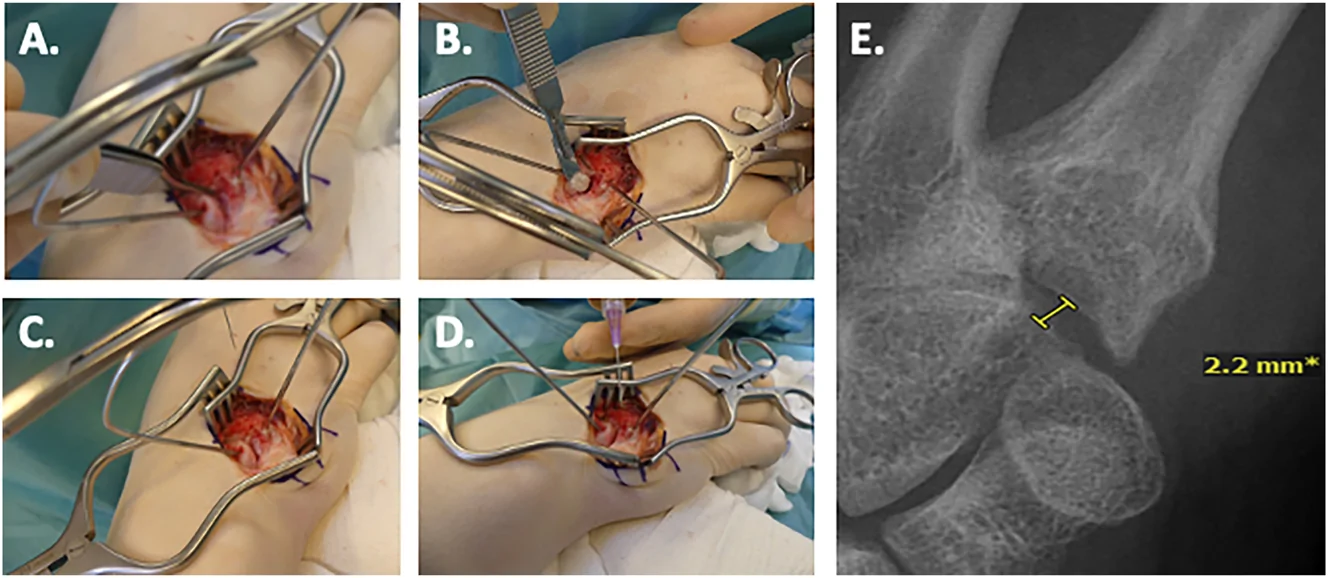
CMC arthroplasty with minced cartilage. (A) Intraoperative view after joint debridement, with forceps indicating the CMC-V joint. (B) Cartilage fragments mixed with PRP prior to transplantation into the cartilage lesion. (C) Transplanted minced cartilage at the base of metacarpal V. (D) Application of thrombin solution to secure the cartilage graft. (E) Postoperative X-ray image of the CMC joint (joint space measured at 2.2 mm).

First, a joint debridement was performed, smoothing the cartilage edges and removing the osteophytes (Figure 2A, with forceps indicating the CMC V joint). The AutoCart™ system from Arthrex (Arthrex Inc., Naples, FL, USA) was used to produce an autologous cartilage graft according to the manufacturer’s instructions. In brief, the AutoCart™ kit was used to collect venous blood, which was then centrifuged to produce platelet-rich plasma (PRP) and converted into a thrombin solution. Autologous cartilage from a non-weight-bearing area of the ipsilateral hamate bone was harvested and minced as described elsewhere [8]. The cartilage fragments were mixed with PRP at a 3:1 ratio and processed into a fragment paste that filled 80–90 % of the cartilage lesion (Figure 1B). Figure 1C shows the cartilage graft inserted into the defect of the CMC V joint. The fragment paste was then carefully covered, drop by drop, with the thrombin solution (Figure 1D). The combination of fibrinogen in the fragment paste with the applied thrombin created a stable clot to hold the mixture within the lesion. For final sealing, PRP was mixed with the thrombin solution in a 1:1 ratio and applied drop by drop. After suturing the CMC V joint capsule and layered wound closure, the wrist was immobilized in a plaster splint for 2 weeks, followed by occupational therapy mobilization for an additional four weeks. Clinical and radiological follow-up examinations at 6 weeks, 6 months and 12 months, showed radiologically normal joint conditions, with a joint space measured at 2.2 mm and no progression of osteoarthritic changes (Figure 1E). One year postoperatively, the patient reported being completely free of pain (VAS 0/10 under stress). Hand function was unrestricted, with full fist closure and intact opposition of all fingers to the thumb. The DASH score improved to 24 points, and grip strength increased to 33 kg compared to 40 kg on the unaffected side. The patient had no complaints or restrictions in axial compression, was subjectively satisfied, and would choose to undergo the procedure again.

## Discussion

To the authors’ knowledge, this case report is the first documented description of treating CMC V osteoarthritis with minced cartilage. The use of minced cartilage could be a promising addition to the treatment options for post-traumatic osteoarthritis of the CMC joints, compared to other approaches like arthroplasty, suspension arthroplasty, or arthrodesis. Long-term results are not yet available. However, initial data on the use of minced cartilage in small joints such as the glenohumeral joint, the metatarsophalangeal joint of the big toe, and the tibiofibular joint, have shown promising results [9], 10]. Additionally, early medium-term results of autologous cartilage transplantation, particularly in the knee joint, suggest that the transplanted cartilage remains viable and that patient satisfaction is high [11]. Although initial clinical findings are encouraging, prospective clinical studies with larger patient populations are required.
